# Managing Chronic Neuropathic Pain: Recent Advances and New Challenges

**DOI:** 10.1155/2022/8336561

**Published:** 2022-10-12

**Authors:** Namrata Hange, Sujan Poudel, Saleha Ozair, Trissa Paul, Meghna Nambakkam, Rakchhya Shrestha, Farrah Greye, Sangam Shah, Yagya Raj Adhikari, Sangharsha Thapa, Pooja Patel

**Affiliations:** ^1^National University of Singapore, Singapore; ^2^National Medical College, Tribhuvan University, Birgunj, Nepal; ^3^Department of Research & Academic Affairs, Larkin Community Hospital, South Miami, FL, USA; ^4^Larkin Community Hospital, Miami, Florida, USA; ^5^Department of Neurology, University of Kansas Medical Center, Kansas City, KS, USA; ^6^Department of Internal Medicine, Ascension St. Agnes Hospital, Baltimore, MD, USA; ^7^Tribhuvan University, Institute of Medicine, Maharajgunj, Kirtipur 44600, Nepal; ^8^University of Minnesota, Minneapolis, MN, USA; ^9^Department of Internal Medicine, Larkin Community Hospital, South Miami, FL, USA

## Abstract

**Aim:**

Neuropathic pain affects 7–10% of the population, with most of the patients receiving inadequate and incomplete treatment. Owing to the high financial burden and the poor quality of life of the patients and their caretakers, there is a dire need to address the challenges in diagnosing and treating chronic neuropathic pain.

**Methods:**

This literature review was conducted to review novel treatments and related challenges through a systematic search from sources such as PubMed, Google Scholar with the combination of MESH words such as neuropathic pain, management of neuropathic pain. Articles from non-English literature, reports without human subjects, animal studies, and abstracts/posters were excluded. However, human studies and studies published in English were included.

**Result:**

This review article discusses novel treatment modalities while acknowledging the challenges medical workers face while encountering neuropathic pain. Despite the recent advances in diagnosis and treatment modalities, several challenges still exist. Hence, there is still a need to explore the various treatment modalities, emphasizing the cause and underlying pathophysiology of neuropathic pain.

**Conclusion:**

We recommend integrated multimodal treatment with the current treatment facility, including various medical disciplines. However, a personalized approach would work the best depending on the 'patient's medical history. Therefore, this article recommends an integrated, cause-specific, cost-effective approach to address this problem of chronic neuropathic pain.

## 1. Introduction

Globally, neuropathic pain is prevalent in 7%–10% of the general population, among which 20–30% have chronicity [1]. Chronic neuropathic pain was redefined in 2011 by the International Association for the Study of Pain (IASP) as: “pain arising as a direct consequence of a lesion or disease affecting the somatosensory system including peripheral fibers” (A*β*, A*δ*, and C fibers) and central neurons [2]. Neuropathic pain has been categorized into a broad range of clinical conditions depending upon etiology (degenerative, traumatic, infectious, metabolic, and toxic) and site of neurological lesion (peripheral vs. central lesion) [2]. Painful peripheral neuropathies, post-herpetic neuralgia, and traumatic nerve injury are all common causes of peripheral neuropathic pain. Positive features, such as aberrant non-painful sensations (e.g., tingling, numbness, pins, and needles) with paresthesia and/or dysesthesia, and negative phenomena, such as neurological sensory deficiencies in the painful area, describe neuropathic pain syndromes clinically [2]. Existing treatment modalities (pharmacological, nonpharmacological, and interventional therapies) essentially provide only symptomatic relief [3]. Pharmacological treatments recommended as the first-line treatment include antidepressants (tricyclic agents, serotonin-norepinephrine reuptake inhibitors) and anticonvulsants (gabapentin and pregabalin). Effective nonpharmacological treatment modalities for chronic pain include behavioral therapy for short-term pain relief; cognitive behavioral therapy for reducing long-term pain and disability; hypnosis as an adjunctive therapy; guided imagery, diaphragmatic breathing, and music therapy for peripheral neuropathy.

Chronic neuropathic pain is associated with a substantial economic burden. It unequally affects females, older ages, and people with low education levels leading to increased labor absenteeism. Compared to other chronic pain, neuropathic pain seems to be more challenging to treat, with the quality of treatment being low as only a few patients receive recommended medication in effective doses [4]. While the total cost of neuropathic pain has not been determined, neuropathic pain boasts substantial costs to society as direct medical costs, reduced ability to work, reduced ability of caregivers to work, and greater need for institutionalization [5]. While pharmacological treatment of neuropathic pain has been widely explored with guidelines such as NICE and NeuPSIG recommendations, inadequate response to such treatment is still a significant unmet need in neuropathic pain patients [6]. There is increased advocacy for developing novel pharmacological approaches addressing the current issue of low efficacy and impaired health-related quality of life [7]. Interventional, psychological, and physical therapy are recommended for drug-refractory cases [8]. Neuropathic pain is also widely underdiagnosed and underrated [9]. Current pharmacological treatments are mainly palliative, and although they temporarily relieve the pain, they do not address the underlying mechanisms of the pain. Over the past two decades, numerous studies on various animal models and extensive clinical research on neuropathic pain have led to a new conceptualization for chronic neuropathic pain syndrome management. Individualized multidisciplinary patient care entails careful consideration of pain-related disability (such as depression and occupational dysfunction) and patient education, as well as repeat follow-up and strategic referral to appropriate medical/surgical subspecialties, as well as physical and psychological therapies [10]. This review summarizes the epidemiology, definition, assessment scales, current treatment options and recent advances, nonpharmacological treatment options, and the challenges of managing these patients. Additionally, the challenges in the diagnosis are also being overviewed.

## 2. Methods

This literature review was conducted to review novel treatments and related challenges through a systematic search from sources such as PubMed, Google Scholar with the combination of MESH words such as neuropathic pain, management of neuropathic pain: nonpharmacological and pharmacological modalities “neuropathic pain,” “neuralgia,” “chronic pain,” “recent advances,” “management,” “treatment,” “pharmacological modalities,” “nonpharmacological modalities.” Articles from non-English literature, reports without human subjects, animal studies, and abstracts/posters were excluded. However, human studies and studies published in English were included. This article aims to review the most recent advances in diagnosing and managing patients with chronic neuropathic pain and the challenges involved in managing patients with chronic neuropathic pain.

## 3. Result and Discussion

Successful Management of chronic neuropathic pain involves identifying the underlying cause and requires an integrated management approach using pharmacological and nonpharmacological approaches.

### 3.1. Diagnosing Chronic Neuropathic Pain (CNP)

Precise diagnostic modalities for chronic neuropathic pain, which consider location and etiology, are crucial for holistic management with improved quality of life, especially in the elderly population [11]. Taking a detailed history should be at the forefront of the diagnostic workup, typically inclusive of location, onset, duration, radiation, characteristics, frequency of pain, intensity, or severity of pain on a scale of 0–10, mode of injury (if any), relieving and aggravating factors. In addition, any associated symptoms, such as decreased range of motion, weakness, stiffness, muscle spasms, and decreased sensation or muscle strength, should be assessed [12]. Autoimmune syndromes like systemic lupus erythematosus or rheumatoid arthritis are common misdiagnoses for peripheral neuropathy, causing a domino effect of incorrect drug treatment and further worsening of the condition.

The physical examination of neuropathic pain includes the examination of cranial nerves (I–XII), muscle strength, the functionality of the autonomic nerves, and light and pin prick (sharp) touch sensations [13].

Laboratory investigations should include testing for vitamin B12 and folate deficiencies, thyroid, liver, and kidney abnormalities, glycosylated hemoglobin, glucose tolerance, lyme disease, hepatitis B and C, celiac disease, anti-MAG antibodies, autoimmune conditions, tox screen, among many others to find a source for neuropathic pain [13]. EMG studies estimate the extent of nerve damage and determine the root cause of neuropathy. A definitive diagnosis would possibly need spinal taps. Radiographic investigations, including computed tomography (CT) and magnetic resonance imaging (MRI), help to diagnose the criticality of disease depending upon the presentation and signs [13]. Another advanced autonomic study, the quantitative sensory test (QST), is used to target certain neuropathies. Small nerve endings which detect temperature and large nerve endings which sense vibrations are checked for damage. This is a non-invasive test regulated by a computer system that recognizes how the isolated nerves respond to vibration and variations in temperature. The results of normal patients and the patient's unaffected side are used as variables to be compared to the patient's affected side to assess for any abnormalities [13]. The most common causes of chronic neuropathic pain can be classified into four categories: trauma, disease, infection, and limb amputation. However, other causes of neuropathic pain include thyroid insufficiency, carpal tunnel syndrome, spinal arthritis, radiculopathies, spondylolisthesis, vitamin B deficiency, and facial nerve palsy [14]. The method-based approach to diagnose neuropathic pain holds tremendous potential for ensuring a better quality of life for patients with neuropathic pain.

### 3.2. Pharmacological Management of Chronic Neuropathic Pain: Recent Advances

**Figure 1:**
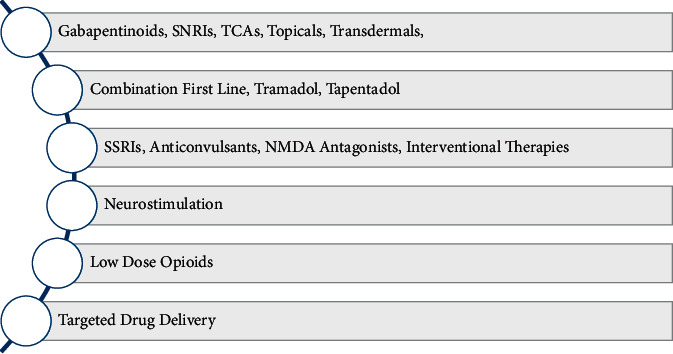
The comprehensive algorithm for the management of chronic neuropathic pain published in 2019.

**Figure 2:**
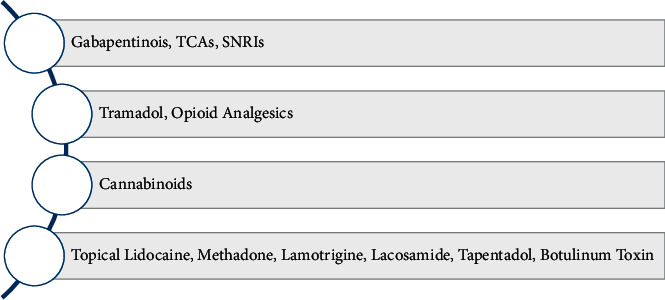
Consensus statement for the management of chronic neuropathic pain by the Canadian Pain Society.

The challenges in the treatment of chronic neuropathic pain are such that despite the availability of various therapeutic regimens, treatment efficacy remains abysmally low. The comprehensive algorithm for the management of chronic neuropathic pain published in 2019 outlines the proposed therapy progression from the first line through the sixth line wherein five of the six phases are mainly pharmacological. A summary of the comprehensive algorithm is presented in Figure 1 [15]. Similarly, the consensus statement for the management of chronic neuropathic pain by the Canadian Pain Society has pharmacologic agents constituting the first through the fourth line of management. A summary of the comprehensive algorithm is presented in Figure 2 [16]. Irving et al. estimated that despite optimization of therapy, only 50% of patients could attain a 30–50% reduction in their pain sensation, while the other half remain refractory to therapeutic management [17]. These dismal statistics leave much to be improved in the domain of pain management in chronic neuropathy. As a result, new pharmacological agents are continually being developed and tested. The study reports recent advances in pharmacological therapy to treat chronic neuropathic pain, not limited to cancer, chemotherapy, or post-surgical causes. These include recently approved drugs, drugs for which clinical trial results have been published, drugs and pharmacological agents undergoing clinical trials, and pharmacological agents in preclinical testing phases.

### 3.3. Approved Drugs

#### 3.3.1. Qutenza (Target: TRPV1 Channel)

An 8% Capsaicin patch (640 mcg per cm^2^; total 179 mg), Qutenza, is a transient receptor potential vanilloid (TRPV1) receptor agonist. Initially approved for the treatment of post-herpetic neuralgia in 2009, a supplemental new drug approval for using Qutenza in the treatment of painful peripheral diabetic neuropathy was released in July 2020 [18]. The drug's efficacy was supported by the results of two 12-week, double-blind, randomized, dose-controlled multicenter clinical trials. In the first study, 29% ± 2% of the treatment group demonstrated a ≥50% reduction in average pain at the primary assessment during week 8 [19, 20]. Only 18% ± 2% of the control group had a ≥50% reduction in average pain at the same assessment point. The second study had comparable results, with 33% ± 2% of the treatment group showing a ≥50% reduction in average pain and a ≥50% reduction in average pain in 26% ± 2% of the control group [17, 20]. The recommended dosage is up to 4 patches applied over the most painful skin areas in an office setting with a minimum interval for repeat dosing as warranted by the return of pain is three months. The most common adverse effects are limited to the application site and include erythema, pain, pruritus, and papules [18].

#### 3.3.2. Mirogabalin (Target: *α*2*δ*1 Subunit of Voltage-Gated Ca^2+^ Channel)

A gabapentinoid therapy, Mirogabalin besylate modulates pain by binding to the *α*2*δ*1 subunit of voltage-gated Ca^2+^ channels in the dorsal root ganglion [21]. Mirogabalin was approved to treat peripheral neuropathic pain in Japan in January 2019. A phase III clinical trial (AMELA) conducted in Japan, Taiwan, and South Korea was completed in December 2020 [22]. Preliminary analysis of the results found that Mirogabalin was superior to placebo when participants were assessed for change in average daily pain score (ADPS) from baseline to week 14 [23]. These results mirrored results from earlier studies, including results from the 14-week, randomized, double-blind, placebo-controlled phase 3 study in Asian patients published in January 2019. At the point of assessment, the difference in average daily pain score least-squares mean vs. placebo in this study was −0.41, −0.47, and −0.77 for Mirogabalin 15, 20, and 30 mg/day, respectively [24]. Subsequently, an extension study assessing the long-term safety and efficacy over 52 weeks was conducted. The results from the extension study showed that adverse effects ranged from mild to moderate and most commonly included nasopharyngitis, somnolence, dizziness, weight increase, and edema [25].

### 3.4. Drugs under Clinical Trials

#### 3.4.1. Ipidacrine (Neiromidine) (Target: Acetylcholinesterase)

A 4-aminopyridine derivative, Ipidacrine is a reversible inhibitor of acetylcholinesterase [26]. A phase II randomized control trial published in 2019 concluded that adding Ipidacrine to the traditional therapy for tunnel syndrome resulted in the regression of neuropathic pain syndrome. This regression was quantitatively assessed using VAS, DN4, and pain detect scales, the indexes of which decreased significantly (*p* < 0.01).

#### 3.4.2. Bumetanide (Target: Na^+^-K^+^-2Cl^−^ Cotransporter)

Bumetanide has been proposed as an adjunctive treatment in the treatment of neuropathic pain resulting from spinal cord injury (SCI). An open-label, single-arm pilot trial of bumetanide (2 mg/day) as an add-on treatment was conducted in 14 SCI patients for 19 weeks. Pain scores were assessed using the numeric rating scale (NRS) and the short-form McGill pain questionnaire. Published in November 2020, the study results demonstrated a significant reduction in the average pain intensity measured by the aforementioned pain scores [27].

#### 3.4.3. Minocycline (Target: Toll-Like Receptor 4)

A tetracycline that is primarily marketed as an antibiotic, minocycline has been shown to alleviate neuropathic pain through microglial inhibition [28]. This is thought to be due to its antagonistic effect on the expression of the toll-like receptor 4, a receptor implicated in the neuroinflammation mediated by microglia [29]. In addition, a placebo-controlled double-blind drug-crossover study demonstrated that [30].

#### 3.4.4. Dietary Agmatine Sulfate

A metabolite of arginine, dietary agmatine sulfate has been proposed by multiple studies as a dietary adjunct in the treatment of refractory chronic neuropathic pain [31]. A pilot open-label consecutive case series study was conducted to demonstrate the effectiveness of agmatine sulfate in patients with painful small fiber neuropathy. Participants showed varied responses with average decrease in pain intensity of 26.0 rating points, corresponding to a 46.4% reduction in overall pain (*p* < 0.00001) [32].

#### 3.4.5. IV Immunoglobulin

Intravenous immunoglobulin is an immune-modulating, blood-derived product that may decrease neuropathic pain by decreasing neuroinflammatory processes [33]. A double-blind, randomized, placebo-controlled, multicenter trial was designed such that 0.4 g/Kg/day, administered intravenously over five consecutive days to the treatment group with primary endpoint assessment of pain intensity and quality of life. Four weeks after IVIG, ≥50% pain reduction was reported in seven of 11 patients (63.6%) in the IVIG group vs. zero of 12 in the placebo group (*P*=0.0013). The only adverse effect reported in the treatment group was mild “dermatitis psoriasiform” in one patient [34].

#### 3.4.6. Topical Cannabidiol Oil

A placebo-controlled, randomized crossover study looking at the effectiveness of topical cannabidiol oil in the symptomatic relief of peripheral neuropathy in the lower extremities found a significant improvement of pain symptoms with the topical application of 250 mg CBD/3fl. oz, as assessed by the neuropathic pain scale. In addition, the treatment group reported a reduction in intense pain, sharp pain, cold, and itchy sensations and no adverse events [35].

#### 3.4.7. Dextromethorphan Post-Ketamine (Target: NMDA-R Antagonist)

Intravenous ketamine is an NMDA antagonist often used to treat refractory neuropathic pain [36]. Though effective, administration of IV ketamine requires trained personnel in a controlled setting. Using an adjunctive oral NMDA antagonist following the procedure would theoretically extend the interval period between office visits. A multicenter randomized controlled clinical trial tested this hypothesis with a three-arm study; dextromethorphan 90 mg/day, memantine 20 mg/day, and placebo. Results collected at one month showed that dextromethorphan-maintained pain relief induced by ketamine while memantine and placebo increased pain intensity scores. Adverse events included drowsiness and nausea in the dextromethorphan group and dizziness, drowsiness, and constipation in the memantine group [37]. Another study compared the anti-nociceptive effects of dextromethorphan to placebo and concluded that because dextromethorphan had no intrinsic anti-nociceptive effect in acute pain on healthy skin, the N-methyl-D-aspartate receptor may need to be sensitized by pain for dextromethorphan to be effective [38].

#### 3.4.8. Micronized Palmitoylethanolamide (Target: Direct PPAR-*α*, Multiple Receptors Indirectly)

Palmitoylethanolamide (PEA) is a neurotrophic-antinociceptive nutraceutical that exerts its effects directly through the PPAR-*α* receptor and indirectly through the TRPV1, CB1, and CB2 receptors [38]. Analysis of a study on micronized PEA in patients with low back pain/sciatica revealed that it significantly reduces the neuropathic pain component of sciatica. The NNT for 600 mg/die of PEA was 1.7 (95% confidence interval: 1.4–2) for pain, and 1.5 (95% confidence interval: 1.4–1.7) for function. PEA was also extremely well-tolerated, with the calculated NNH non-significant [39].

#### 3.4.9. Cetuximab (Target: EGFR-1 Antagonist)

It is hypothesized that cetuximab exerts its effects in reducing neuropathic pain by directly inhibiting MAP-kinase in neuronal or glial cells [40]. Several case series and reports have been published wherein treatment with cetuximab resulted in significant alleviation of neuropathic pain, an open-label randomized proof of concept failed to demonstrate a significant confidence interval for the reduction of pain scores; however, 36% of patients in the treatment group reported ≥50% reduction in average pain three to seven days after cetuximab compared to 14% among the placebo group. This warrants further investigation. The only adverse event reported was grade 1–2 skin rashes among 86% of the participants [41].

#### 3.4.10. Nano Curcumin (Target: Proinflammatory Cytokine Inhibitor)

Curcumin is an antioxidant that inhibits the production of proinflammatory cytokines like TNF-*α* and Interleukin-1 (IL-1) and prevents NO synthesis [42]. A double-blind, randomized, placebo-controlled clinical trial conducted in 2019 concluded that curcumin supplementation for two months improved and reduced the severity of DSPN in patients with T2DM [42]. Supplementation of nano curcumin was accounted for a significant reduction in Glycated hemoglobin (HbA1c) (*p* < 0.001) and fast blood sugar (FBS) (*p*=0.004), total score of neuropathies (*p* < 0.001), total reflex score (*p*=0.04), and temperature (*p*=0.01) compared to placebo group [42].

#### 3.4.11. PF-06372865 (Target: *α*2/*α*3/*α*5 Subtype-Selective GABA_A_ Partial Agonist)

A novel *α*2/*α*3/*α*5 gamma-aminobutyric acid A (GABA_A_) subunit selective partial positive allosteric modulator (PAM) [43]. In a randomized placebo-controlled crossover study published in 2019, researchers found that a dose of 15 mg PF-06372865 enhanced pain tolerance thresholds (PTTs) for pressure pain by a ratio of 1.11 (90% confidence interval [CI]: 1.02, 1.22) when compared to placebo [43]. PTT for the cold pressor increased by 1.17 (90% CI: 1.03, 1.32), and pressure pain task increased by 1.11 (90% CI: 1.01, 1.21) after a dose of 65 mg PF-06372865. [43]

#### 3.4.12. Topical Fisionerv® (Target: Multimechanistic NeuP Modulator)

An emulgel obtained from the combination of Carbopol 990 Polymer (which gels water) and Carbopol Ultrez 20 (which emulsifies ozonated olive oil) [44]. A double-blind, randomized controlled clinical trial was conducted in 2019 on patients with lumbar sciatica or lumbar disk herniation and/or lumbar canal stenosis, or with carpal tunnel syndrome with two treatment arms: group A: in Fisionerv® in combination with physiotherapy and group B: placebo in combination with physiotherapy. A significant (*p* < 0.05) improvement was observed group A (VAS mean 5.3 (1.10)) compared to group B (VAS mean 6.17 (0.80)), already after four weeks of treatment [44]. A further VAS reduction was recorded at eight treatment weeks, with a significant difference between the treatments (group A: VAS mean = 1.89 (0.77); group B: VAS mean = 3.79 (1.20) (*p* < 0.001) [44]. No adverse drug reaction was observed [44].

#### 3.4.13. Lacosamide (Target: Na Channel Blocker)

The anticonvulsant lacosamide specifically acts on Nav1.3, Nav1.7, and Nav1.8 which play a significant role in the modulation of neuropathic pain in small fiber neuropathy [45]. The lacosamide-efficacy-“N”-safety in SFN (LENSS) randomized, placebo-controlled, double-blind, crossover-design study was carried out between November 2014 and July 2016 [45]. It showed that lacosamide has a significant effect on pain, general wellbeing, and sleep quality [45]. In 58.3% of patients receiving lacosamide, mean average pain decreased by at least 1 point, compared to 21.7% in the placebo group [sensitivity analyses, odds ratio 5.65 (95% confidence interval: 1.83–17.41); *P*=0.0045] [45]. In the lacosamide group, 33.3% reported that their general condition improved versus 4.3% in the placebo group (*P*-value = 0.0156) [45]. Lacosamide was well-tolerated and safe, suggesting that it can be used for pain treatment in Nav1.7-related small-fiber neuropathy [45].

#### 3.4.14. Intrathecal Baclofen (Target: GABA Analogue)

A GABA analogue that has analgesic effects on all subtypes of neuropathic pain and can improve interference of neuropathic pain with activities of daily living [46]. In a randomized controlled trial conducted in 2018, neuropathic pain was proven to be improved significantly in the intrathecal baclofen (ITB) group based on assessments such as the numerical rating scale, brief pain inventory, and neuropathic pain system inventory, which revealed an effect on all subtypes of pain [46].

#### 3.4.15. Sustained Release Sodium Nitrite

Oral formulation of NaNO_2_ improves vascular function by restoring NO bioavailability and mediating vasodilation resulting in potential effectiveness as a therapeutic agent in diabetic peripheral neuropathy and endothelial dysfunction [47]. In a double-blind, randomized placebo-controlled clinical trial conducted in 2018, 24 patients were randomized to 40 mg or 80 mg of sustained-release nitrite or placebo twice daily for 12 weeks [47]. The NPSI assessment of patients in the 40 mg and 80 mg dose group reported a 12.7% and 22.0% reduction in pain, respectively, compared to an 8.4% reduction by patients in the placebo group [47]. In addition, SR-nitrite eliminates the headache and dizziness observed in the immediate release of nitrite [47].

#### 3.4.16. *α*-Lipoic Acid

Lipoic acid, a potential antioxidant, improves diabetic neuropathy by improving blood flow, reducing oxidative stress, and improving distal nerve conduction [48]. A prospective interventional study conducted in 2015–2016 concluded that lipoic acid administration was associated with reduced neuropathic symptoms and improved quality of life in painful diabetic neuropathy patients [49]. Patients noted statistically significant reductions in neuropathic symptoms following a 40-day administration of 600 mg a-lipoic acid, indicated by changes in NSS, SPNSQ, and DN4 scores. [49]

#### 3.4.17. Lidocaine Infusion

Lidocaine, a widely used local anesthetic and class Ib antiarrhythmic, has effectively treated neuropathic pain [50]. A prospective, randomized, double-blind, placebo-controlled, parallel study concluded that patients with lidocaine infusion therapy (3 mg/kg of lidocaine administered over 1 hour once a week for four weeks) showed a significant percentage reduction in NRS pain scores after the final infusion compared to the control group [50]. However, the pain reduction was not maintained at the 4-week follow-up [50].

#### 3.4.18. l-4 Chlorokynurenine (AV-101)

AV-101 (l-4 chlorokynurenine): it is a novel oral agent which is converted into a potent, selective GlyB site antagonist of the NMDAR, and demonstrated to be active in neuropathic pain [51]. The randomized, double-blind, placebo-controlled two phase 1 studies conducted in 2017 concluded that although AV-101 did not reach statistical significance in reducing pain, there were consistent reductions for allodynia pain and mechanical and heat hyperalgesia. [51] The excellent pharmacokinetic and safety profile warrants further clinical trials [51].

#### 3.4.19. Turpentine Oil

Turpentine oil is a safe topical analgesic and skin protectant observed to relieve neuropathic pain [52]. A randomized control trial conducted in 2016 concluded that patients treated with commercially available turpentine oil had a VAS of 7.83 ± 1.012 at baseline and 5.20 ± 1.187 after three months of treatment (*P*-value 0.0001) [52].

#### 3.4.20. Dronabinol

Dronabinol is a purified active compound of medical cannabis, which acts primarily upon CB_1_ and CB_2_ receptors resulting in a wide range of medical indications, including analgesia [53]. A randomized, double-blind, placebo-controlled parallel-group design for 16 weeks was followed by a 32 weeks open-label period [53]. In addition, a subgroup of patients participated in the open-label long-term safety follow-up for up to an additional 96 weeks [53]. This trial demonstrated that dronabinol is a safe long-term treatment option as there were no reported signs of drug abuse with only one possible case of dependency [53].

#### 3.4.21. Neublastin

Neublastin targets proteins that belong to a group of glial cell-derived neurotrophic factors that act on sensory neurons and assist in the regeneration of sensory nerve cells relieving neuropathic pain [54]. Study designs consisted of clinical trials reporting minimal pain relief with the lowest injection dose of 50 *μ*g/kg for lumbosacral radiculopathy [54]. Approximately 50% of the non-placebo group reported pruritus unrelated to any specific doses [54].

#### 3.4.22. Botulinum Toxin A

It binds to presynaptic cholinergic nerve terminals and decreases the release of acetylcholine, causing neuromuscular block [55]. A randomized controlled trial reflects significant relief in neuropathic pain with subcutaneous injections [55]. Complaints of pain during injection administration for 30% were the only side effect noted [55].

#### 3.4.23. Cebranopadol

This opioid alternative analgesic that combines nociceptin/orphanin FQ peptide (NOP receptors) receptor reduces afferent neuronal excitability, a critical pathway for pain transmission [56]. The clinical trial with higher doses of 400–600 *μ*g had 50% of participants with pain relief (3/10) but had high dropout rates in trials with chronic low back pain [56]. In addition, higher doses of 400–600 *μ*g reported adverse effects during titration and discontinuation of the drug [56]. Dizziness, nausea, vomiting, somnolence, constipation, fatigue, headache, and hyperhidrosis were common side effects [56].

#### 3.4.24. BG00010 (Neurotrophic Factor) (Target: GFR*α*3 Co-Receptor)

A protein in the glial cell line-derived neurotrophic factor (GDNF) family, BG00010 is a selective ligand for the GDNF family receptor alpha-3 (GFR*α*3) co-receptor [57]. This receptor normalizes cellular changes resulting from damage or disease and potentially alleviates neuropathic pain [57]. A randomized, blinded, placebo-controlled multiple-dose study in subjects with sciatica showed that higher dose regimens of BG00010 resulted in a more significant pain reduction than placebo or lower dose regimens. The most adverse effects were mild and included headache, feeling hot, and pruritus [57].

#### 3.4.25. Sativex®

Sativex, a combination of delta-9-tetrahydrocannabinol (THC) and cannabidiol (CBD), may be effective in improving MS-related neuropathic pain [58]. This is possible through its action on specific cortical pathways. A small-scale clinical trial assessed the clinical (such as VAS) and neurophysiological (using laser-evoked potentials and transcranial magnetic stimulation) parameters before and after four weeks of Sativex administration [58]. The study showed that one month of drug administration in MS patients with neuropathic pain successfully reduced pain rating and improved quality of life [58].

#### 3.4.26. Topical Ketamine (Target: N-Methyl-D-Aspartate Antagonist)

The assessment of the efficacy and safety of an alternative route of administration of ketamine, an open-label trial, showed that subjects who applied topical ketamine 10% three times a day for two weeks had a decrease in their numerical pain scale by the end of two weeks, ranging from 14% to 63% [36]. No adverse effects were reported in the trial [59].

### 3.5. Nonpharmacological Management of Chronic Neuropathic Pain: Recent Advances

Nonpharmacological pain management methods for chronic neuropathic pain proved to be valuable in a multimodal approach, combined with pharmacotherapy concurrently or separately [60, 61].

#### 3.5.1. Neurostimulation and Neuromodulation Techniques

Although usually invasive, neurostimulation techniques provide effective pain control in patients with chronic neuropathic pain due to paraplegia and above-knee amputation [60, 62]. Neuromodulation therapies can positively impact underlying disease mechanisms and move from a palliative treatment paradigm to a disease prevention model. Neuromodulation is a non-addictive, drug-free clinical tool for treating chronic neuropathic pain [62]. It works by sending controlled physical energy to pre-identified neural targets in the central nervous system, either by actively stimulating nerves to produce a natural biological response or by applying targeted pharmaceutical agents in tiny doses directly to the site of action. Neuromodulation for pain is divided into peripheral and CNS modulation, as well as invasive and non-invasive interface types [62]. Electrodes are applied to the brain, spinal cord, or peripheral nerves in neurostimulation devices.

#### 3.5.2. Epidural Motor Cortex Stimulation (EMCS)

It is known to be a safe, efficacious, therapeutic option for chronic neuropathic and drug-resistant pain. Neurosurgeons specifically prefer it for such cases [63]. In addition, surgically-implanted EMCS has emerged as an alternative in the advanced management of facial neuropathic pain and is proven to relieve refractory neuropathic pain effectively [63].

#### 3.5.3. Spinal Cord Stimulation (SCS)

It was a commonly used invasive technique for pain relief of various neuropathic pain conditions. It works through the stimulation of the dorsal columns of the spinal cord by administering electrical impulses at frequencies of approximately 50 Hz (by an implanted pulse generator-PG) and the electrodes (or lead). It can suppress the central neuronal hyperexcitability with a remarkable reduction of pain intensity scores. Despite being well known for a relatively lower side effect profile such as excessive sedation or cognitive impairment, SCS has been associated with serious adverse events such as infection, dural puncture leading to a subdural hematoma, and subsequently death in one of the patients [64, 65]. Nevertheless, SCS is considered a valid, effective, and safe treatment option in patients suffering from neuropathic pain and those resistant to pharmacological treatment. Technical advancements to improve the clinical efficacy and novel waveform paradigms, such as Burst-SCS and KHFSCS (Kilohertz-frequency spinal cord stimulation)—a novel paresthesia-free treatment for chronic pain—have contributed significantly towards innovations in lead design, improvements in IPG capabilities to increase battery lifetime, reduce invasiveness, and provides closed-loop stimulation. These innovations and decades of clinical experience have produced potentially powerful therapies to improve the lives of patients suffering from chronic pain. Nevertheless, the level of efficacy for SCS in chronic pain is still classified as “moderate” and with minimal side effects. In case of failure of SCS in pain management or central origin pain, clinicians switch to deep brain stimulation or motor cortex stimulation [66].

#### 3.5.4. Deep Brain Stimulation (DBS)

It is used to treat neuropathic pain in post-peripheral nerve injury, post-spinal cord injury, brain stem disease and thalamic pain, pain after amputation, either phantom or stump, cranial and facial pain, including anesthesia dolorosa, and plexopathies. DBS trials with lead externalization have been used extensively over the last 50 years to treat chronic pain. However, spinal cord and/or peripheral nerve stimulation have overshadowed DBS in the pain arena. Laser thermal ablation and radiosurgery provide cost-effective, efficacious therapy and an ablation-based alternative to DBS, possibly used with magnetic resonance imaging guidance to resolve neuropathic pain, especially in advanced stages of cancer [67].

#### 3.5.5. Peripheral Nerve Stimulation

It has been proven to be helpful in the treatment of several chronic pain conditions, including peripheral nerve dysfunctions, complex regional pain syndrome, and cranial neuralgias. These peripheral nerve stimulators allow external pulse generators to transmit impulses wirelessly to the implanted electrode, and their implantation is lesser invasive. Lately, TIMWAVE, Bioness, and SPR Therapeutics have produced a new generation of devices that allow for external pulse generators to transmit impulses wirelessly to the implanted electrode. In a study conducted by Mobbs et al., significant improvement in symptoms and lifestyle quality was reported post-implantation of peripheral nerve stimulators; [68] while Deer et al. has demonstrated significant relief for severe intractable chronic pain with the application of an externally powered peripheral nerve stimulator in a prospective, multicentric, randomized, double-blinded, partial crossover study [69]. In addition, PNS utilizing percutaneously inserted electrodes was shown to be effective in occipital neuralgia, trigeminal neuropathic pain, and cervicogenic headache [70–74].

#### 3.5.6. Transcutaneous Electrical and Electromagnetic Stimulation

Transcutaneous electrical nerve stimulation (TENS) is an expensive, non-invasive, self‐adhesive to relieve pain in CNP, PNP, complex regional pain syndrome, phantom limb pain, and fibromyalgia [75]. The administered technique delivers pulsed low voltage electrical current.

#### 3.5.7. Electroceuticals

These devices deliver therapeutic electrical stimulation, often at very high frequencies, to different parts of the body. Techniques for chronic pain are being developed, including epidural stimulation and vagal nerve stimulation, which can be quite invasive and expensive. In addition, the analgesic mechanisms of TENS can be evaluated with advanced neuroimaging techniques, such as electroencephalography, magnetoencephalography, and magnetic resonance imaging [76].

#### 3.5.8. Repetitive Magnetic Stimulation

The use of implanted epidural stimulation to treat persistent, drug-resistant neuropathic pain has been proposed. Various investigations have shown that recurrent transcranial magnetic stimulation (rTMS) administered to the motor cortex can reduce neuropathic pain, at least temporarily [77]. Additionally, adjuvant static magnetic field therapy can significantly decrease exercise-induced pain in patients with diabetic polyneuropathy (level II evidence).

#### 3.5.9. Transcranial Direct Current Stimulation (tDCS)

It is a recently developed, non-invasive, painless brain stimulation treatment that uses direct electrical currents to stimulate specific brain parts. MRI-guided transcranial focused ultrasound is a nascent, minimally invasive method of targeted tissue thermal ablation that may be used to treat central neuropathic pain with current and potential intracranial applications. Transcranial magnetic-resonance-imaging-guided focused ultrasound is a very precise and a radiation-free neurosurgical technique for treating neuropathic pain [78]. In addition, adjuvant repetitive transcranial magnetic stimulation of the motor cortex effectively reduces the overall pain intensity in patients with diabetic polyneuropathy (level II evidence).

#### 3.5.10. Remote Electrical Neuromodulation (REN)

Remote electrical neuromodulation has proven to be a novel acute treatment modality for migraine by stimulating the upper arm peripheral nerves to induce conditioned pain modulation (CPM) [79]. This technique utilizes an endogenous analgesic mechanism in which conditioning stimulation inhibits pain in remote body areas.

### 3.6. Novel Innovative Techniques

#### 3.6.1. Immersive Virtual Reality (IVR)

Therapy, an emerging technique, has not been made widely available yet as the equipment is expensive and possibly challenging for clinicians, especially those with limited technological experience. However, with the development of “plug and play,” low-cost IVR devices such as the Oculus Rift, Gear VR, and Google Cardboard, IVR no longer requires such specific technical proficiency. Therefore, IVR may be a feasible and affordable option for adjuvant therapy of neuropathic pain. A preliminary study was conducted to review the application of a new virtual reality method and 3D augmented reality mirror visual feedback therapy in persistent, unilateral upper extremity neuropathic pain, and a significant reduction of pain was reported [80].

#### 3.6.2. Breathing Controlled Electrical Stimulation (BreEStim)

It is a novel, nonpharmacological intervention for neuropathic pain wherein a single-pulse electrical stimulation (EStim) was delivered transcutaneously to a peripheral nerve study.

#### 3.6.3. Scrambler Therapy (Marketed as Calmare™ Therapy in the United States)

It is a novel form of superficial neuromodulation, electro-analgesia therapy for non-invasive pain relief in chronic neuropathic and cancer pain, based on a new generation of medical devices that use a rapidly changing electrical impulse to send a “non-pain” signal along the same pain fibers that are sending the “pain” stimulus. This is predicted to potentially treat chronic neuropathic pain and cancer pain resistant to opioids. Scrambler Therapy was created to trap C-fibers and relieve neuropathic pain, but it has also been shown to help with numbness and tingling caused by various nerve fibers [81, 82].

#### 3.6.4. Optogenetics

It is an upcoming alternative strategy to reduce pain using genetically encoded and optically active proteins, opsin, to control signaling events by directly manipulating somatosensory pathways. Evidence from animal models shows that optogenetics is an efficient approach to alleviating inflammatory and neuropathic pain. Considering that gene therapy techniques are already at the clinical stage for several pathological conditions, analgesia by phototherapy could be a promising strategy to treat chronic pain [83].

#### 3.6.5. Photon Stimulation or Pulsed Infrared Light Therapy

Management of patients with painful diabetic peripheral neuropathy (PDPN) is done by photon stimulation delivered using LEDs. Photo biomodulation or low-level laser therapy (LLLT) utilizes electromagnetic technology, which significantly improves the quality of pain and QOL outcomes in patients with a significant amount of pain and disability due to diabetes [84]. Furthermore, the application of photo biomodulation in nerve injury and pain control has also been associated with a decrease in inflammatory cytokine levels and facilitation of neuronal regeneration, as demonstrated by the levels of TNF-a, IL-1b, and GAP-43 [85].

3.6.6. Vibration Stimulation: (WBV) has recently been shown to improve strength, balance, endurance, and blood and lymph fluid circulation in patients with stroke and peripheral neuropathy associated with Parkinson's disease [86]. WBV improves sensory perceptions like pain and vibration, neuropathy disability score, balance measures, and the general health in painful diabetic peripheral neuropathy [87]. Vibration causes local myorelaxation, which behaves like a massage and enhances the overall analgesic effects [86].

#### 3.6.6. Molecular Modalities

Proposed molecular modalities for treating chronic neuropathic pain include ion channel expressions, immune reactions, and inflammatory substrate diffusions. Recent advances in RNA sequence analysis have discovered specific ion channel expressions in nociceptors such as transient receptor potential (TRP) channels, voltage-gated potassium, and sodium channels. G protein-coupled receptors (GPCRs) also play an essential role in triggering the surrounding immune cells. Multiple protein expressions are thought to complicate the therapeutic development of neuropathic pain. Recent progress in optogenetics and pharmacogenetics may herald the development of novel therapeutics for incurable pain. Optogenetics and designer receptors exclusively activated by designer drugs (DREADDS) provide hope in controlling intractable pain in patients suffering from debilitating neuropathic pain. Based on the underlying cause of neuropathic pain, it may be possible to improve pain management by using a combination of therapies and drugs targeting specific molecular targets. With continued development and refinement of this technique combined with viral vector-mediated safe and efficient gene therapy, there is a hope to develop a satisfactory treatment for otherwise intractable neuropathic pain [88].

#### 3.6.7. Possible Stem Cell Regenerative Therapeutic Dimension with Transplantation

This is a new approach to repair damaged nervous system-induced neuropathic pain syndromes rather than simply providing palliation. Stem cells offer a totipotent cellular source for replacing injured or lost neural cells. Stem cells have the ability to stop degenerative processes, suppress apoptotic pathways, and let wounded and uninjured nerves survive and regenerate. The ability of stem cells to change cellular processes, when combined with their ability to release neurotrophic factors, creates a protective and restorative milieu that has the potential to reverse the source of neuropathic pain. Stem cells can play many functions in the damaged microenvironment, such as peripheral, central, and spinal cord disinhibition, which greatly reduces clinical symptoms including spontaneous pain and hyperalgesia [89–91]. The proposed mechanism replaces the damaged neuronal tissue, protects against progressive nerve damage, and releases paracrine and endocrine factors, which repair the underlying pathology of genesis and propagation of damage within the somatosensory system. Previous studies have shown that fetal GABAergic neuron precursors can also provide substantial pain relief. Lastly, GABAergic neuron transplants have been found to survive long-term in an injured spinal cord, which can possibly treat neuropathic pain along with synaptic integration.

#### 3.6.8. Oxygen and Ozone Therapy

O_2_, O_3_ therapy is considered a promising conservative and mini-invasive intervention that can be used independently or in combination with other treatments to reduce neuropathic pain and improve pain function in patients affected by musculoskeletal disorders such as low back pain and knee osteoarthritis [92].

#### 3.6.9. Aptiva

Aptiva is an innovative non-drug option that uses a frequency rhythmic electrical modulation system (FREMS) to treat painful diabetic neuropathy. It uses modulated electrical pulses, which differ from those used in percutaneous electrical nerve stimulation (PENS), and it is intended to improve the supply of oxygen to nerve cells to reduce pain. However, current evidence regarding efficacy is limited due to the high costs of this type of therapy. Possibly because of underlying huge cost [93].

#### 3.6.10. Cognitive Behavioral Therapy (CBT)

A biopsychosocial approach used in several multidisciplinary pain management programs for reducing the severity of pain associated with diabetic peripheral neuropathy. CBT encourages patients to take control of their pain and lead a fulfilling life despite it [94]. Addressing the psychological component of chronic neuropathic pain is central to success. CBT strategies comprise 8 to 12 sessions, lasting 30 to 50 minutes, including psychoeducation, relaxation, cognitive restructuring, stress management, activity pacing, behavioral activation, anger control, and relapse prevention techniques [95].

#### 3.6.11. Complementary and Integrative Medicine (CIM)

This includes nonconventional treatments such as acupuncture, meditation, massage, cupping, movement therapies, and relaxation techniques that may be used in conjunction with or in place of other medical treatments.

#### 3.6.12. Acupuncture

A minimally invasive procedure using traditional Chinese therapy based on real experiences has proven beneficial and cost-effective in managing diabetic peripheral neuropathy, back pain, and shoulder pain [96, 97]. This involves the stimulation of specific points on the body, most often by inserting thin needles through the skin, promoting the body's natural self-healing process. Acupuncture also stimulates blood flow to restore nerve damage. Acupuncture was found to be an effective treatment option for diabetic and human immunodeficiency virus-related peripheral neuropathy, especially in Bell's palsy and carpal tunnel syndrome [98–100]. Battlefield acupuncture (BFA) is an emerging method of easing neuropathic pain, purported to influence central nervous system pain processing through the release of *β*-endorphins and its effect on a somatotopic organization of the body represented in the auricle.

#### 3.6.13. Aromatherapy

One of the most widely used complementary therapies in clinical practices involves the use of essential oils extracted from plants that reduce neuropathic pain scores and improve the QoL, resulting in a positive impact on physical, emotional, and mental health [101]. Preclinical research studies have reported that the essential oil obtained from bergamot (BEO) fruit (Citrus bergamia, Risso) modifies the normal and pathological synaptic plasticity implicated, for instance, in nociceptive and neuropathic pain. In addition, BEO modulates the sensitive perception of pain in different models of nociceptive, inflammatory, and neuropathic pain modulating endogenous systems.

### 3.7. Challenges in Managing Chronic Neuropathic pain

A good treatment strategy might take several trials of combining pharmacological and nonpharmacological therapeutic options. Pain management counseling and frequent communication is a must to target the right fit of regimes. The best outcomes have shown improvement over time with experimentation, much like a sliding scale for pain management.

#### 3.7.1. Tolerability and Safety

The most common adverse effects of various therapeutic options discussed above were constipation, nausea, drowsiness, and dizziness. An increase in the frequency of serious adverse events occurred with chronic treatment and polypharmacy.

#### 3.7.2. Precautions

Precautions are warranted in patients with a history of substance abuse and other significant comorbidities like heart disease and dementia. A dosage adjustment is essential in renal failure or CYP450 enzyme-inhibitor interactions.

#### 3.7.3. Noncompliance and Quality of Life

Inadequate pharmacological response significantly contributes to drug non-compliance. In the context of neuropathic pain, minimal to no relief of pain can potentially lead to sleep disturbance, depression, fatigue, reduction in quality of life and driving ability.

## 4. Limitations

1. Many of the newer drugs that we mentioned in the study are in phase I and II. So, these drugs are not approved for use.
2. We could not formulate new treatment modalities for the treatment of neuropathic pain
3. Our study does not include articles comparing superiority of combining pharmacological and nonpharmacological treatment modalities over pharmacological.
4. Disease-specific treatment modalities are not clear.
5. Limited articles on recent advances in psychological treatment modalities.

## 5. Conclusion

The increasing prevalence of chronic neuropathic pain and its poor impact on the quality of life prompts the need for an early diagnosis and intervention. Recent advances in various pharmacological modalities have been primarily limited by the side effect profile of certain drugs and the cost incurred in making them available to the masses. Research addressing nonpharmacological measures might help to come up with cost-effective solutions with minimal side effects. A mixed approach consisting of symptomatic management during an acute episode and a simultaneous multidisciplinary approach for long-term pain management is thus warranted.

## Data Availability

All the data used to support the findings of the study are included within the article.
